# Correlation of AHR Activation-Induced Suppression of PWM-Stimulated IgG and IgG-Triggered Signaling in Human PBMCs

**DOI:** 10.3390/antib15040068

**Published:** 2026-08-04

**Authors:** Arpita Deb, Barbara L. F. Kaplan

**Affiliations:** Center for Environmental Health Sciences and Department of Comparative Biomedical Sciences, College of Veterinary Medicine, Mississippi State University, Mississippi State, MS 39762, USA; ad2368@msstate.edu

**Keywords:** aryl hydrocarbon receptor (AHR), indole-3-carbinol (I3C), IgG

## Abstract

Background/Objectives: Aryl hydrocarbon receptor (AHR) ligands are known to suppress antibody production. However, it remains unclear whether this suppression translates into altered antibody-mediated Fcγ receptor signaling in human cells. This study aimed to investigate the effects and mechanisms by which AHR ligands regulate antibody production and antibody-triggered responses in human peripheral blood mononuclear cells (PBMCs). Methods: PBMCs were isolated from blood obtained from anonymous healthy donors. PBMCs were stimulated with pokeweed mitogen (PWM) to induce IgG1 production or Strept-Biotin IgG1/IgG2 immune complexes to induce antibody-triggered signaling. Before any stimulation, cells were pretreated with vehicle (0.01% DMSO) or AHR ligands TCDD (2,3,7,8-tetrachlorodibenzo-p-dioxin), ITE (2-(1H-indol-3-ylcarbonyl)-4-thiazolecarboxylic acid methyl ester), or FICZ (6-formylindolo[3,2-b]carbazole), or the proligand I3C (indole-3-carbinol). Results: Our data revealed that PWM stimulation significantly increased *IL6* and *IL1B* gene expression and PWM-induced IL-6 cytokine secretion, which was significantly suppressed by TCDD, ITE, FICZ, and I3C. However, only TCDD was able to suppress PWM-stimulated IgG1 antibody production. Transcriptomic analysis using I3C revealed upregulated AHR-responsive genes such as *CYP1A1*, *CYP1B1*, *AHRR* and *TIPARP* in PWM-stimulated human PBMCs. Notably, I3C downregulated genes *TRAPPC9* and *C1QTNF3*, which play a role in NF-κB-associated inflammatory signaling. It also revealed potential sex differences in I3C-mediated gene modulation associated with B cell function, Ig expression, FcγR signaling, and inflammatory pathways. Lastly, IgG1 and IgG2 immune complex-stimulated IL-6 cytokine secretion was significantly suppressed by TCDD, whereas I3C showed modest, although not statistically significant, suppression. Conclusions: Overall, these findings demonstrate that AHR activation by TCDD suppressed antibody production and IgG-mediated immune signaling in human PBMCs.

## 1. Introduction

The aryl hydrocarbon receptor (AHR) is a ligand-activated transcription factor that has emerged as an important regulator of immune responses [1]. 2,3,7,8-tetrachlorodibenzo-p-dioxin (TCDD) is the prototypical AHR ligand that exhibits potent immunosuppression, including suppression of antibody production, cytotoxic T lymphocyte (CTL) activity, and delayed hypersensitivity responses [2,3]. Antibody-mediated immune responses are essential in the host defense system; however, they can also contribute to inflammation and autoimmunity. Among the five primary types (isotypes) of immunoglobulins, immunoglobulin G (IgG) is the most abundant subclass of antibody in serum. Previous epidemiological studies have demonstrated an inverse relationship between circulating IgG levels and TCDD exposure in humans [4,5,6,7]. Many in vitro studies have also demonstrated AHR ligand-mediated modulation of IgG levels in both animal model and human systems [8,9,10,11,12,13]. Despite these findings, the mechanisms underlying AHR-mediated suppression of antibody production are not fully understood, and the mechanism of action is not necessarily conserved across species. In addition, there is a significant data gap that exists in determining the effect of AHR ligands on total IgG and IgG subtypes.

Antibodies also play a critical role in immune effector functions through Fcγ receptors. Antibodies can form an immune complex and engage FcγRs on the surface of immune cells. This causes clustering of the receptors and triggers the activation of downstream signaling pathways that mediate a range of immune responses [14]. The human FcγR family is comprised of four subclasses (FcγRI; FcγRIIA, FcγRIIB, and FcγRIIC; FcγRIIIA and FcγRIIIB; and FcRn) [15]. Activation of FcγR by antibodies can trigger immune responses such as phagocytosis, degranulation, antibody-dependent cellular cytotoxicity (ADCC), cytokine production, and release of inflammatory mediators [16,17]. While TCDD-mediated suppression of antibody production has been documented, how the compromised IgG production by AHR activation might be translated into antibody-triggered downstream signaling in humans is not well understood.

In this study we aimed to characterize the effects and underlying mechanisms by which AHR-activating chemicals alter IgG and the consequences of AHR activation-induced alteration of IgG antibody production in human peripheral blood mononuclear cells (PBMCs). Besides TCDD, we examined the effects of endogenous AHR ligands 2-(1H-indol-3-ylcarbonyl)-4-thiazolecarboxylic acid methyl ester (ITE) and 6-formylindolo[3,2-b]carbazole (FICZ), and a natural dietary proligand, indole-3-carbinol (I3C). By combining functional assays and transcriptomic analysis, this study aimed to provide mechanistic insight into how AHR activation influences IgG and IgG-mediated signaling in human PBMCs.

## 2. Materials and Methods

Human PBMCs: Human blood leukopaks from anonymous, healthy donors were purchased from the Gulf Coast Regional Blood Center (Houston, TX, USA). Whole blood samples (approximately 50 mL) were diluted with 1× phosphate-buffered saline (PBS) and layered over Histopaque 1077 (Millipore/Sigma; St. Louis, MO, USA) for density-gradient separation. Differential centrifugation separated the plasma, buffy coat layer containing PBMCs, and red blood cells and granulocytes. After PBMCs were isolated from the buffy coat, cells were washed. Cell viability and total live-cell counts were determined using an acridine orange/propidium iodide (AO/PI) viability dye. Cells were plated in a U-bottom 96-well plate at either 1 × 10^6^ live cells/mL in 100 μL per well for immune complex stimulation (24 h) or at 0.5 × 10^6^ live cells/mL in 200 μL per well for pokeweed mitogen (PWM), which required longer culture duration. Complete medium consisted of 1× RPMI supplemented with 5% bovine calf serum (BCS) and 1% penicillin–streptomycin.

PWM stimulation and chemical treatment: PBMCs were seeded on day 0, and the cultures were maintained for an additional 2 or 5 days following PWM stimulation, which occurred on day 1. We initially conducted time optimization experiments with PWM and determined that IgG production was donor-dependent and was induced at days 5, 7 and 9 post-PWM stimulation (Table S1). For potential transcriptomic changes that might influence IgG production at day 5, we selected an earlier timepoint; in this case, day 2 post-PWM stimulation. PBMCs were treated with vehicle (VH) control (0.01% DMSO) or AHR-activating chemicals: TCDD (30 nM), ITE (1 µM), FICZ (0.1 µM), or I3C (2 µM) in 0.01% DMSO in 200 µL volume on day 0. AHR-activating chemical concentrations were based in part on solubility using the highest concentration that could be delivered in 0.01% DMSO. After overnight incubation, the cells were stimulated with 5 µg/mL PWM (lectin from *Phytolacca americana*; Millipore Sigma, St. Louis, MO, USA). On day 2 post-PWM stimulation, cells and supernatants were harvested for downstream analyses. For the longer 5-day stimulation, 100 µL of spent medium was removed and replaced with 100 µL of fresh complete medium on day 2 post-PWM stimulation. Cells were harvested on day 2 post-PWM stimulation for RNA-Seq analysis and supernatants were harvested on days 2 or 5 post-PWM stimulation for IL-6 or IgG1 ELISA analyses, respectively.

IgG complex stimulation and chemical treatment: PBMCs were seeded on day 0, and the cultures were maintained for an additional 24 h following IgG complex stimulation, which occurred on day 1. Human IgG1 or IgG2 immune complexes were generated using a streptavidin-biotin assembly approach following our previously published immune complex protocol developed for mouse antibodies [18,19]. Streptavidin (Strept; 4 µg; Thermo Scientific, Waltham, MA, USA) was combined with biotinylated human IgG1 or IgG2 (1 µg; Human IgG1, Ancell Corporation, Bayport, MN, USA; Human IgG2, Sino Biological, Paoli, PA, USA) in 1× PBS and tumbled end over end for 3 h at 37 °C (total volume of 110 µL, from which 100 µL was added to culture for stimulation). PBMCs were pretreated with either VH (0.01% DMSO), TCDD (30 nM), or I3C (2 µM) in 0.01% DMSO in a 100 µL volume on day 0. After an overnight incubation, cells were treated again with chemicals to account for the additional 100 µL volume for the addition of the IgG immune complexes, immediately followed by stimulation by adding 100 µL/well of Strept-Biotin IgG1 or Strept-Biotin IgG2 immune complex overnight. Supernatants were harvested at 24 h post-IgG complex stimulation for cytokine ELISA analyses or cells were used for flow cytometry analysis.

RNA isolation and cDNA synthesis: PBMCs from day 2 post-PWM stimulation culture were harvested, and total RNA was isolated from the cell pellet using the RNeasy mini-Kit (Qiagen; Germantown, MD, USA) according to the manufacturer’s instructions. RNA was quantified by Nanodrop (Thermo Fisher; Waltham, MA, USA), and samples were normalized to an equivalent RNA concentration using RNase-free water. Complementary DNA (cDNA) was generated by reverse transcription using the High-Capacity cDNA Reverse Transcription Kit (Applied Biosystems/Thermo Fisher) with equal amounts of total RNA per reaction. Reverse transcription was performed on a 2720 Thermal cycler (Applied Biosystems) using the following cycling conditions: 10 min at 25 °C, 120 min at 37 °C, and 10 s at 85 °C, followed by a 4 °C hold.

Quantitative Real-Time PCR (qRT-PCR): Synthesized cDNA was used for qRT-PCR to assess *IL6* and *IL1B* gene expression. *IL6* was quantified using a TaqMan assay kit (Applied Biosystems), and *IL1B* was quantified using a SYBR Green PCR kit (Qiagen). Amplifications were performed on an AriaMx Real-Time PCR System (Agilent; Santa Clara, CA, USA). For *IL6*, reactions were assembled in a final volume of 20 µL containing 4 µL cDNA, 1 µL TaqMan Gene Expression Assay for human *IL6* (Hs00174131_m1; Thermo Fisher), 1 µL TaqMan assay for the 18S rRNA endogenous control (Applied Biosystems), and 10 µL TaqMan Universal Master Mix (Applied Biosystems/Thermo Fisher). Cycling conditions were: 95 °C for 10 min, followed by 40 cycles of 95 °C for 30 s, 55 °C for 1 min, and 72 °C for 30 s. For *IL1B*, SYBR Green reactions were prepared in a final volume of 20 µL containing 7.2 µL cDNA, 10 µL QuantiNova SYBR Green PCR Master Mix (Qiagen), 1.4 µL forward primer (1036401814, Hs-IL1b-F; Eurofins Genomics, Lancaster, PA, USA), and 1.4 µL reverse primer (1036401813, Hs-IL1b-Rev; Eurofins Genomics). The housekeeping gene GAPDH was amplified in separate SYBR Green reactions using the same master mix and cycling program with forward primer (1046296648, GAPDH-F; Eurofins Genomics) and reverse primer (1046296649, GAPDH-Rev; Eurofins Genomics). SYBR cycling conditions were: 95 °C for 2 min, followed by 40 cycles of 95 °C for 5 s and 60 °C for 10 s (combined annealing/extension), and a dissociation (melt) curve was performed at the end of amplification at 95 °C for 30 s, 65 °C for 30 s, and 95 °C for 30 s. Gene expression levels were expressed as fold change using the ΔΔCt method with either 18s rRNA or GAPDH housekeeping genes as the endogenous reference.

ELISA analysis: Supernatants from PWM-stimulated PBMC cultures were harvested on days 2 or 5 post-PWM stimulation and assessed for human IL-6 or IgG1 secretion, respectively, by ELISA. Supernatants from immune complex-stimulated PBMC cultures harvested at 24 h post-IgG complex stimulation were assayed for human IL-6 production by ELISA. While we also analyzed the IgG complex-stimulated supernatants for TNF-α and IL-1β, they were not detected and will not be discussed further. Human IgG1 levels were quantified using a pre-coated Human IgG1 ELISA kit (Invitrogen/Thermo Fisher) according to the manufacturer’s instructions. Human IL-6 was measured using a matched antibody pair following standard sandwich ELISA procedures. For all ELISAs, signals were developed using horseradish peroxidase avidin, with subsequent incubation with 3,3′,5,5′-tetramethylbenzidine (TMB) substrate. The enzymatic reaction was stopped with 2N sulfuric acid (H_2_SO_4_), and absorbance was measured at 450 nm using a SpectraMax M5 microplate reader (Molecular Devices, Sunnyvale, CA, USA).

Flow cytometry: Cells from female or male PBMCs treated with AHR-activating chemicals and IgG2 immune complexes were stained with a Zombie NIR fixable viability kit (BioLegend, San Diego, CA, USA) to select live cells, followed by incubation with APC anti-human CD14 (BioLegend) to identify monocytes within the PMBCs. Cells were analyzed on a NovoCyte (ACEA Biosciences, Inc./Agilent, Santa Clara, CA, USA). Fluorescence minus one (FMO) and single-stain cell (SSC) controls were included to guide the gating strategy. Data were analyzed using the NovoExpress software version 2.2.0 (Agilent).

Sample preparation for RNA-sequencing: PBMCs were seeded and pretreated with VH (0.01%DMSO) or the AHR proligand I3C (2 µM) on day 0 and stimulated the next day with PWM for an additional 2 days. Cells were seeded across 10 wells per condition at 0.5 × 10^6^ live cells/mL, yielding a combined total of 5 × 10^6^ cells per sample. On day 2 post-PWM stimulation, cells were harvested and rinsed with ice-cold 1× PBS to remove residual medium. Cell pellets were sent to Novogene (Sacramento, CA, USA) on dry ice for RNA isolation, RNA quality assessment, cDNA library preparation, and Illumina RNA-seq. Reads were paired-end, with 150 base pairs at a depth of >20 million reads. Reads were assessed for quality. Data from high-throughput sequencing platforms (i.e., Illumina) was transformed into sequenced reads (raw data or reads) by CASAVA base recognition (base calling). Raw data were stored in FASTQ (fq) format files. The sequencing reads/raw reads often contain low-quality reads or reads with adapters and therefore were processed to remove adapter-contaminated reads and low-quality bases (base quality score < 5). Clean reads were aligned to the human GRCh38 reference genome (NCBI) using HISAT2 software version 2.2.1.

Transcriptomic analysis: Differentially expressed genes (DEGs) were analyzed using DESeq2 software version 1.20.0 in NovoMagic 2025 (Novogene). DGEs with a padj ≤ 0.05 and ∣log2FoldChange∣ ≥ 1 were considered significant. Female and male donors were identified by screening for expression of Y chromosome-specific genes. The four comparisons by sex and treatment were performed to identify differentially expressed genes. Genes of interest were additionally curated using the Gene Filtering function within NovoMagic.

Functional analysis: Functional enrichment analysis was conducted in NovoMagic Gene Ontology (GO) and Reactome databases. GO is a database that describes human gene functions and their relationship to diseases. GO enrichment was evaluated across the three GO domains (Biological Process, Cellular Component, and Molecular Function). The Reactome database compiled various reactions and biological pathways of model organisms, including humans. Pathways with a padj ≤ 0.05 were considered significant.

Statistics: Data are presented as the mean ± SEM. Statistical analyses were performed using nonparametric a Wilcoxon matched-pair signed rank test or nonparametric Kruskal–Wallis test followed by Dunn’s post hoc test for multiple group comparisons. Significance was defined at *p* < 0.05. Percent and fold-change data were transformed before ANOVA. Data were normalized to a VH within each donor and combined to present all data. All data were subjected to Grubbs’s outlier test, and a single outlier was removed when detected. Raw data are provided in Table S1.

## 3. Results

### 3.1. Effects of AHR-Activating Chemicals on PWM Stimulation in Human PBMC

**Figure 1:**
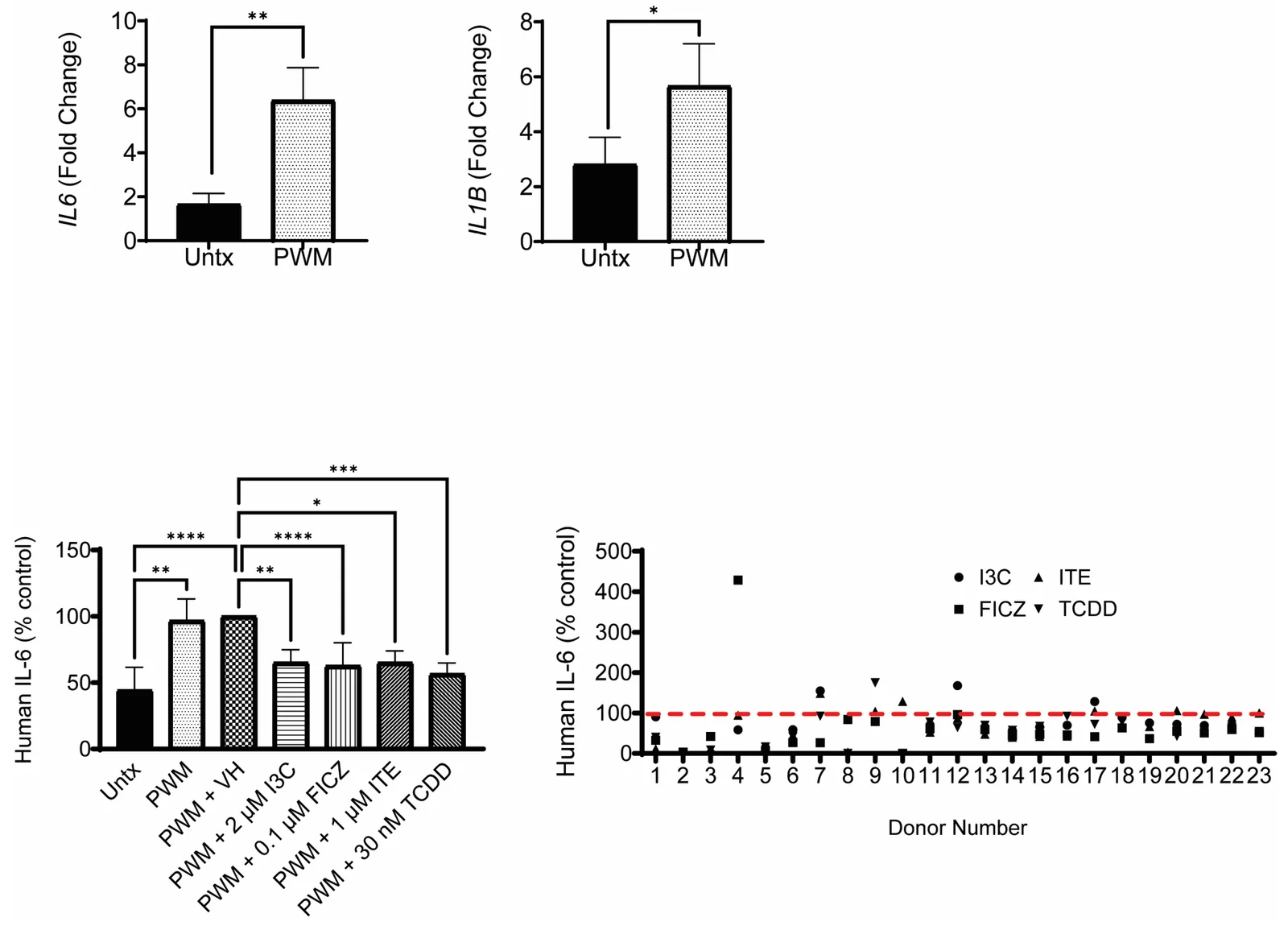
PWM upregulated *IL6* and *IL1B* gene expression and AHR-activating chemicals suppressed IL-6 cytokine secretion 2 days after PWM-mediated stimulation. Human PBMCs (mixed sex; sex was not known at time of culture) were plated at 0.5 × 10^6^ live cells/mL in 200 μL per well. Cells were pretreated with either 0.01% DMSO or AHR ligand TCDD (30 nM), ITE (1 µM), FICZ (0.1 µM), or proligand I3C (2 µM) in 0.01% DMSO overnight. Cultures were stimulated with PWM the next day and then incubated for 2 days. Cells of the Untx and PWM group were analyzed for *IL6* and *IL1B* gene expression by qRT-PCR to confirm PWM stimulation. Supernatants from the culture were assessed for IL-6 cytokine secretion via ELISA. Data obtained in pg/mL were normalized within each donor so data could be combined across donors. qRT-PCR results are from *N* = 24 donors and ELISA results are from *N* = 23 donors; red line indicates the PWM + VH control set to 100%. Before analysis, all datasets were screened for outliers using Grubbs’s test, and a single outlier was removed when detected. Data are mean ± SEM. * indicates statistical differences compared to the PWM + VH control at *p* < 0.05 *, *p* < 0.01 **, *p* < 0.001 *** or *p* < 0.0001 ****.

We first confirmed that PWM effectively stimulated human PBMCs. Human PBMCs were stimulated with PWM for 2 days, after which cells were harvested, and proinflammatory gene expression was quantified by qRT-PCR. PWM stimulation resulted in a significant upregulation of the proinflammatory cytokine genes *IL6* and *IL1B* compared to untreated (Untx) control cells (Figure 1). Once we confirmed the cellular activation of PBMC by PWM, we next evaluated whether AHR-activating chemicals modulated PWM-induced cytokine production. Culture supernatants collected at day 2 post-PWM stimulation were analyzed for IL-6 secretion as one cytokine that is also involved with antibody secretion. Relative to PWM + VH, all AHR-activating chemicals significantly reduced PWM-induced IL-6 release from human PBMCs (Figure 1). We also examined the AHR activation-induced suppression of IL-6 by donor.

### 3.2. Transcriptome Profile of AHR Proligand Treatment

**Figure 2:**
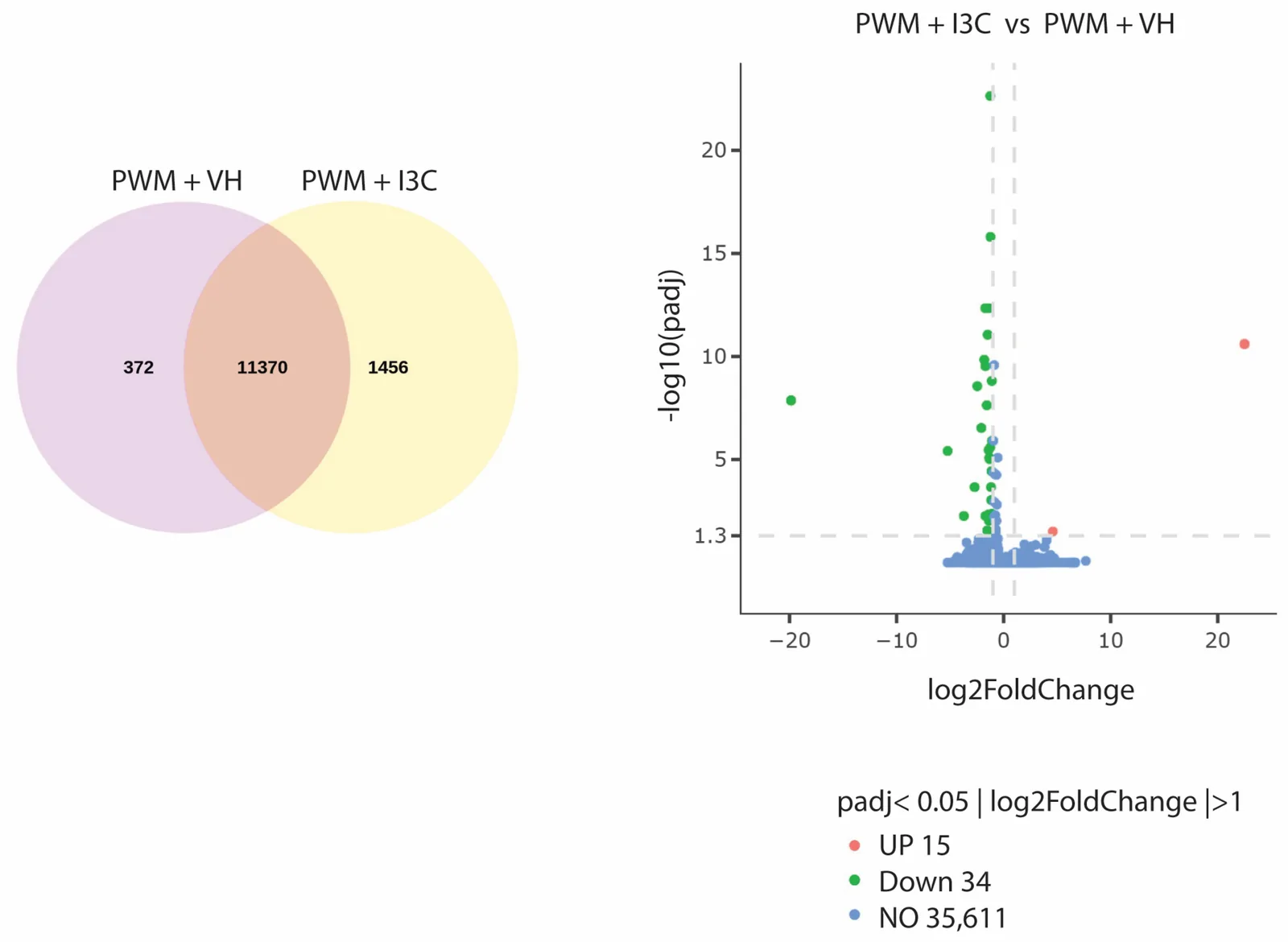
Transcriptomic profile of I3C treatment on PWM-stimulated human PBMCs. PBMCs treated with VH (0.01% DMSO) or I3C (2 µM) were stimulated with PWM for 2 days and analyzed using RNA-Seq. The Venn diagram shows commonly expressed genes in PWM + VH and PWM + I3C groups; the volcano plot shows the upregulated and downregulated genes in PWM + I3C with DEG analysis. Results are from *N* = 15 donors. DEGs were analyzed using DESeq2 software in NovoMagic. DEGs with a padj ≤ 0.05 and ∣log2FoldChange∣ ≥ 1 were considered significant. Dashed lines represent the threshold cutoffs.

While TCDD is the most potent AHR ligand, its toxicity limits its ability to be developed into an immunosuppressive therapy. Therefore, we focused on nontoxic AHR ligands, in particular the dietary proligand I3C since it has exhibited potential to be anti-inflammatory and immune-suppressive [20,21,22]. To define the transcriptional impact of I3C with PWM treatment, we performed RNA-seq on PBMCs treated with PWM in the presence of VH or I3C from 15 total healthy donors (5 male, 10 female). Across all donors, approximately 11,370 genes were detected as expressed in common. Using a significance threshold of padj  <  0.05 and log2(FC)  >  ∣0.5∣, 49 genes were identified as differentially expressed in response to PWM + I3C treatment compared to PWM + VH, with 15 genes upregulated and 34 genes downregulated (Figure 2). Table 1 shows the downregulated genes from the DEG analysis in PWM + I3C-treated cells with some information on their various functions.

### 3.3. Functional Analysis

**Figure 3:**
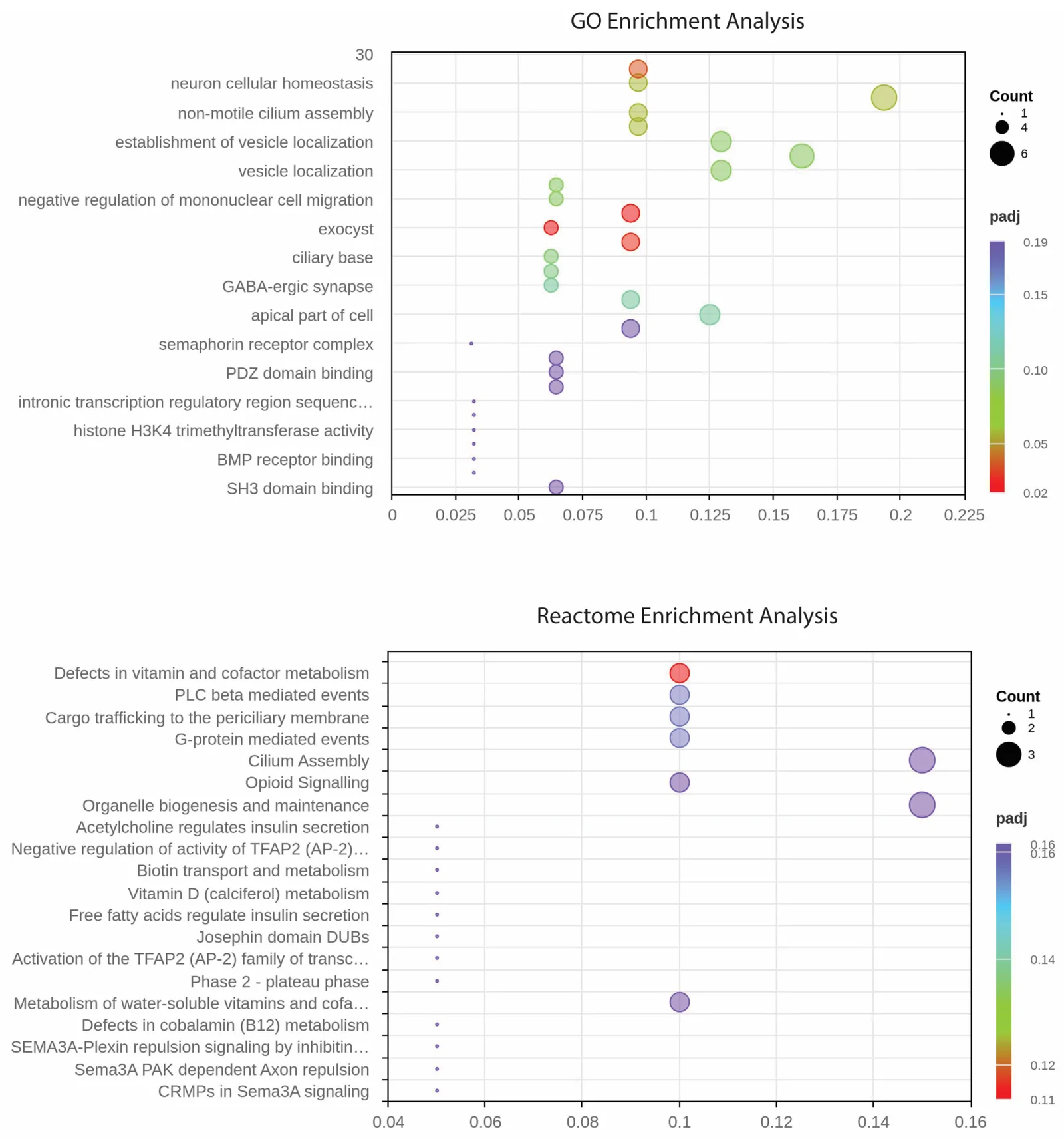
Pathway enrichment analysis of I3C treatment on PWM-stimulated human PBMCs. PBMCs treated with either VH (0.01% DMSO) or I3C (2 µM), followed by stimulation with PWM for 2 days, were analyzed using RNA-Seq. Data were analyzed using NovoMagic software. Results are from *N* = 15 donors. padj ≤ 0.05 was considered as the threshold for significant enrichment in GO term and Reactome analysis. Truncated titles: in GO, intronic transcription regulatory region sequence-specific DNA binding; in Reactome, negative regulation of activity of TFAP2 (AP-2) family transcription factors, activation of the TFAP2 (AP-2) family of transcription factors, metabolism of water-soluble vitamins and cofactors, and SEMA3A-Plexin repulsion signaling by inhibiting integrin adhesion.

DEG genes were enriched for GO function and Reactome function. GO term analysis across all donors revealed enrichment of biological processes, cellular components, and molecular functions. With PWM + I3C treatment, there was a total of 1249 GO terms downregulated, with 996 categorized as biological process, 106 as cellular component, and 147 as molecular function. Figure 3 shows the 10 most affected GO terms, which include neuron cellular homeostasis, negative regulation of mononuclear cell migration, and GABAergic synapse. Reactome pathway enrichment analysis was also performed on identified DEGs. The analysis revealed enrichment of pathways associated with PLCβ-mediated events, G protein-mediated signaling, cargo trafficking to the periciliary membrane and organelle biogenesis and maintenance.

### 3.4. AHR Pathway Activity in PBMCs in Response to PWM + I3C

**Figure 4:**
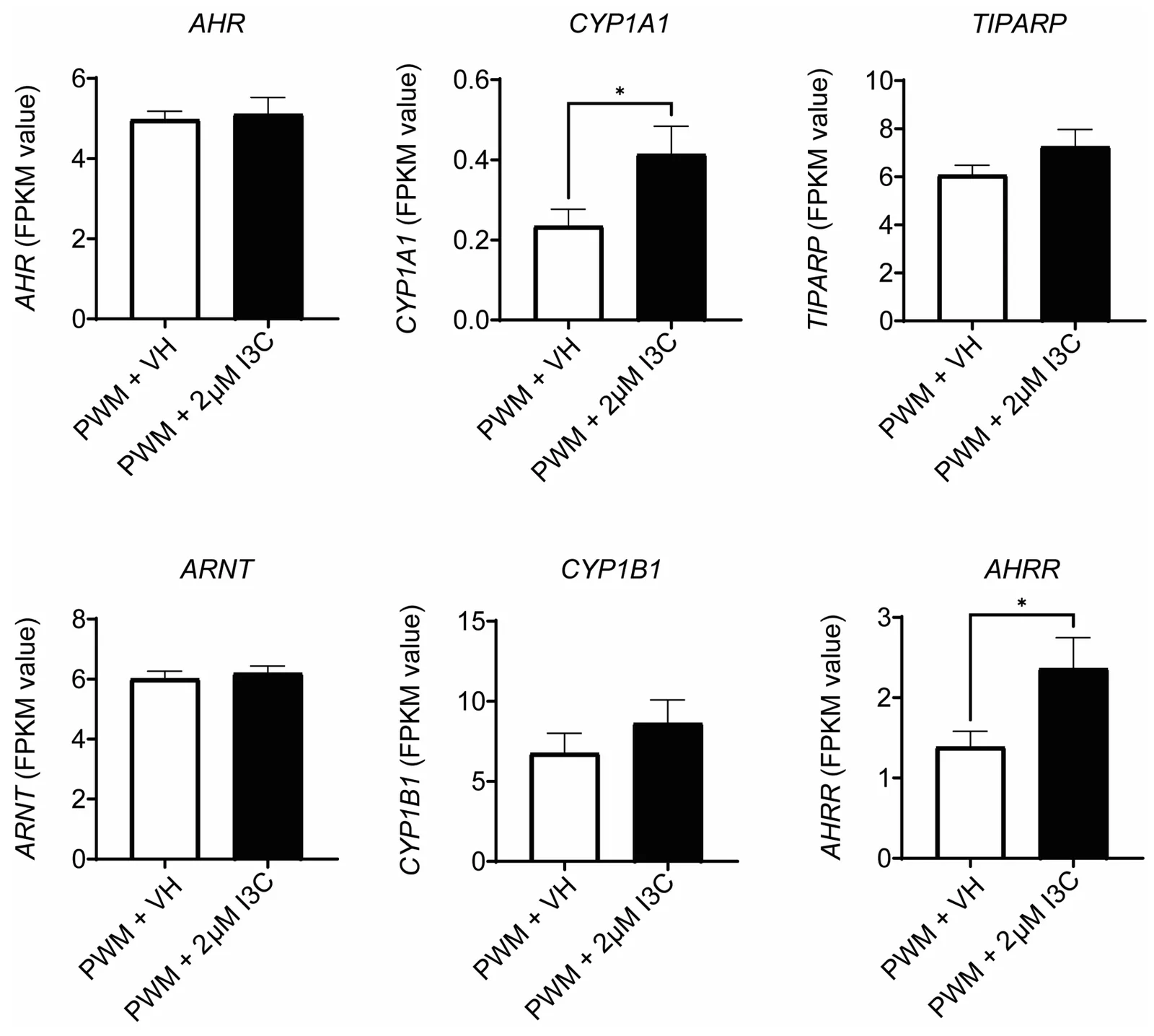
I3C-mediated AHR pathway activation in PWM-stimulated human PBMCs. PBMCs treated with either VH (0.01% DMSO) or I3C (2 µM), followed by stimulation with PWM for 2 days, were analyzed using RNA-Seq. Results are from *N* = 15 donors. Data are expressed as FPKM values. * indicates statistical differences compared to the control PWM + VH group at *p* < 0.05.

To assess whether I3C induced a canonical AHR transcriptional signature, we examined expression levels of *AHR*, *ARNT*, *CYP1A1*, *CYP1B1*, *AHRR*, and *TIPARP* using the Fragments Per Kilobase of transcript per Million mapped reads (FPKM value) across all donors. Expression of *AHR* and *ARNT* remained largely unchanged in PWM + I3C as compared to PWM + VH. In contrast, *CYP1A1* was significantly upregulated, and *CYP1B1* showed a modest, although not statistically significant, upregulation in the PWM + I3C group. Additionally, the AHR negative-feedback regulators *AHRR* and *TIPARP* were induced following PWM + I3C exposure, with *AHRR* significantly upregulated, but not *TIPARP* (Figure 4).

### 3.5. PWM + I3C-Mediated Gene Regulation and Its Potential Association with B Cell Function, Immunoglobulin Expression, Fcγ Receptor Signaling, and Inflammatory Responses

**Figure 5:**
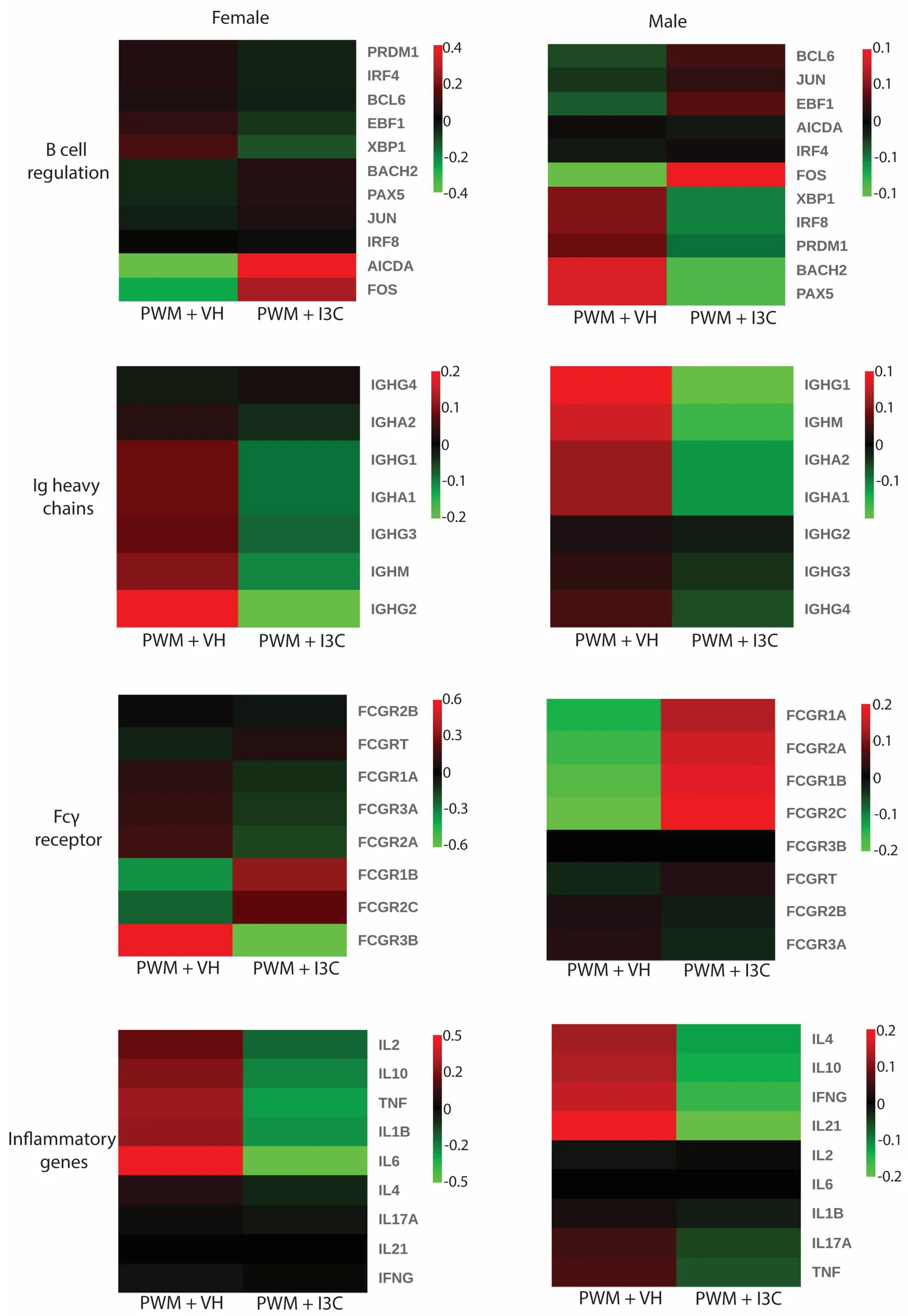
I3C-mediated profiling of genes involved in B cell regulation, Ig heavy chains, Fcγ receptor genes, and inflammatory genes in female and male donors. PBMCs treated with either VH (0.01% DMSO) or I3C (2 µM), followed by stimulation with PWM for 2 days, were analyzed using RNA-Seq. Heatmaps represent the FPKM value of the genes, with rows being normalized using Z-scores. Results are from *N* = 5 male; *N* = 10 female donors. Heatmaps were generated using the hierarchical cluster analysis tool in NovoMagic software. The color of each square reflects the value obtained after normalizing the row expression data; it does not reflect the actual gene expression value. Red indicates upregulation; green indicates downregulation.

Next, to determine whether PWM + I3C treatment influenced genes related to B cell regulation, Ig heavy chains, inflammatory responses, or FcγR signaling compared to PWM + VH, FPKM values were analyzed for genes within these functional categories. To account for potential sex-specific differences, human donors were separated based on the expression of Y chromosome-specific genes: *UTY*, *DDX3Y*, *KDM5D*, *TMSB4Y*, *EIF1AY*, and *RPS4Y1*. This enabled the separation of the donors into male and female groups. Figure 5 shows heatmaps illustrating the gene expression patterns. It should be noted that the overall effects in PWM + I3C versus PWM + VH are quite modest and not statistically significant, with the observed z-scores substantially lower than the typical significant threshold (usually between −2 and 2) associated with a strong differential expression pattern. Nevertheless, these heatmaps represent relative differences between the PWM + VH and PWM + I3C groups but could reveal some sex-specific trend differences that can be assessed in more detail in future studies. In female donors, *AICDA* and *FOS* were upregulated following PWM + I3C treatment, whereas in male donors, only *FOS* expression was increased. In contrast, several key transcriptional regulators of B cell differentiation and function, including *XBP1*, *IRF8*, *PRDM1*, *BACH2*, and *PAX5*, were downregulated in male donors. Genes encoding Ig heavy chains such as *IGHG1*, *IGHA1*, *IGHM* were downregulated in both male and female donors following PWM + I3C treatment. PWM + I3C-mediated effects on inflammatory gene expression were variable and sex-specific. Proinflammatory cytokine genes *IL2*, *IL10*, *TNF*, *IL1B*, and *IL6* were downregulated in female donors and *IL4*, *IL10*, *IFNG*, and *IL21* expression was downregulated in male donors. Interestingly, FcγR genes expression also demonstrated sex-specific regulation. In male donors, *FCGR1A*, *FCGR2A*, *FCGR1B*, and *FCGR2C* were upregulated following PWM + I3C treatment, whereas in female donors, only *FCGR1B* and *FCGR2C* showed modest upregulation. However, *FCGR3B* expression was downregulated in female donors.

### 3.6. Effects of AHR-Activating Chemicals on Human IgG1 Secretion

**Figure 6:**
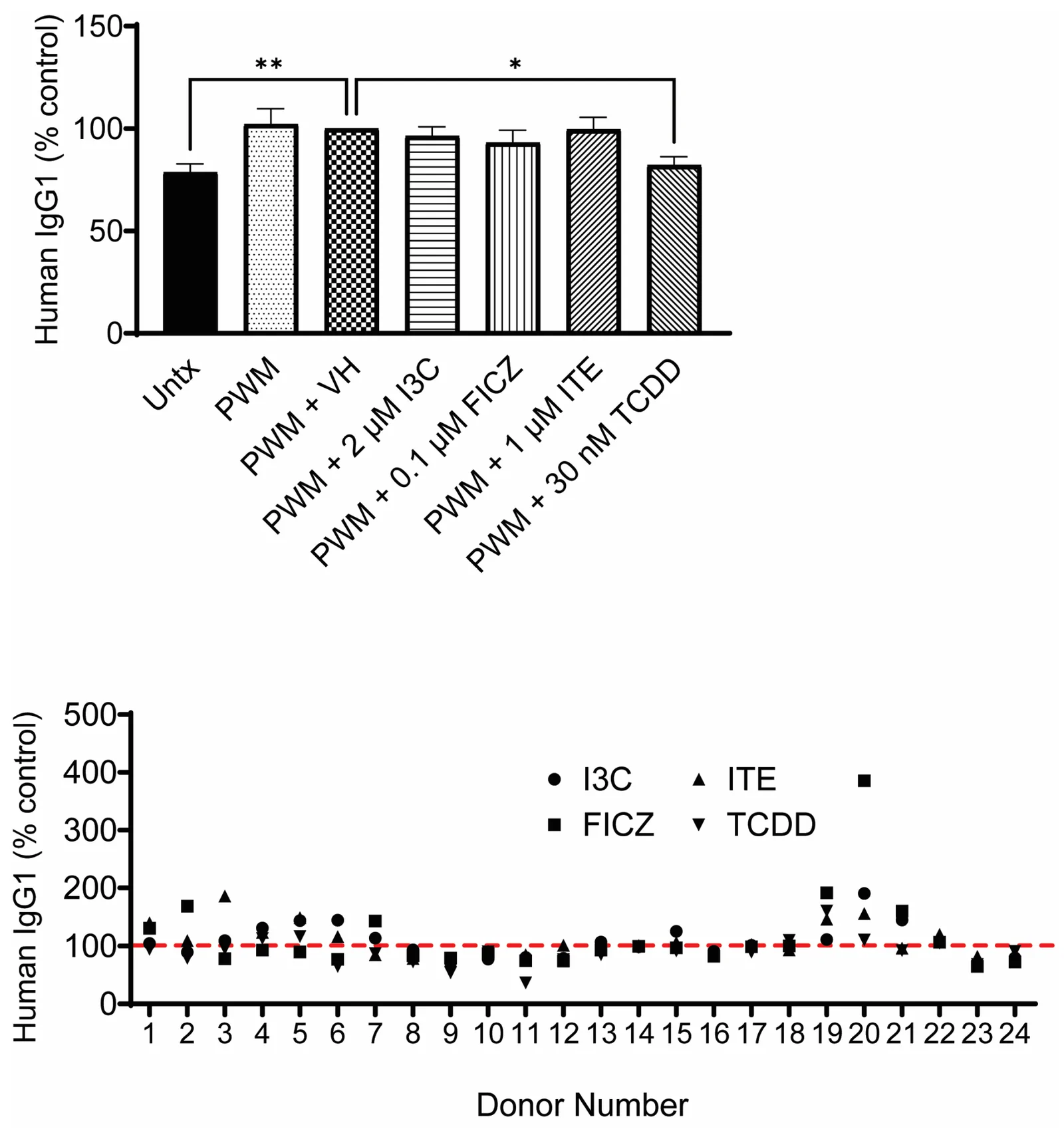
AHR ligand TCDD suppressed IgG1 production 5 days after PWM-mediated stimulation. Human PBMCs (mixed sex; sex was not known at time of culture) were pretreated with VH (0.01% DMSO) or AHR ligand TCDD (30 nM), ITE (1 µM), FICZ (0.1 µM), or the proligand I3C (2 µM) in 0.01% DMSO overnight. Cultures were stimulated with PWM the next day and then incubated for 5 days, replacing half of the media at day 2 after PWM stimulation. Supernatants from the culture were assessed for IgG1 secretion via ELISA. Data obtained in ng/mL were normalized within each donor so data could be combined across donors. Results are from *N* = 22 donors; red line indicates the PWM + VH control set to 100%. Before analysis, all datasets were screened for outliers using Grubbs’s test, and a single outlier was removed when detected. Data are mean ± SEM. * indicates statistical differences compared to the PWM + VH control at *p* < 0.05 * or *p* < 0.01 **.

We next evaluated whether AHR-activating chemicals modulated PWM-induced IgG1 production by human PBMCs. At day 5 post-PWM stimulation, it was determined that only TCDD significantly suppressed IgG1 production. When data were separated out by donor, some donors were sensitive to AHR activation-induced suppression of IgG1, suggesting that there are donor-dependent effects of PWM-driven IgG1 production in response to various AHR ligands or the proligand I3C (Figure 6).

### 3.7. AHR-Activating Chemicals Suppressed IgG1 and IgG2 Antibody-Triggered Cytokine Production

**Figure 7:**
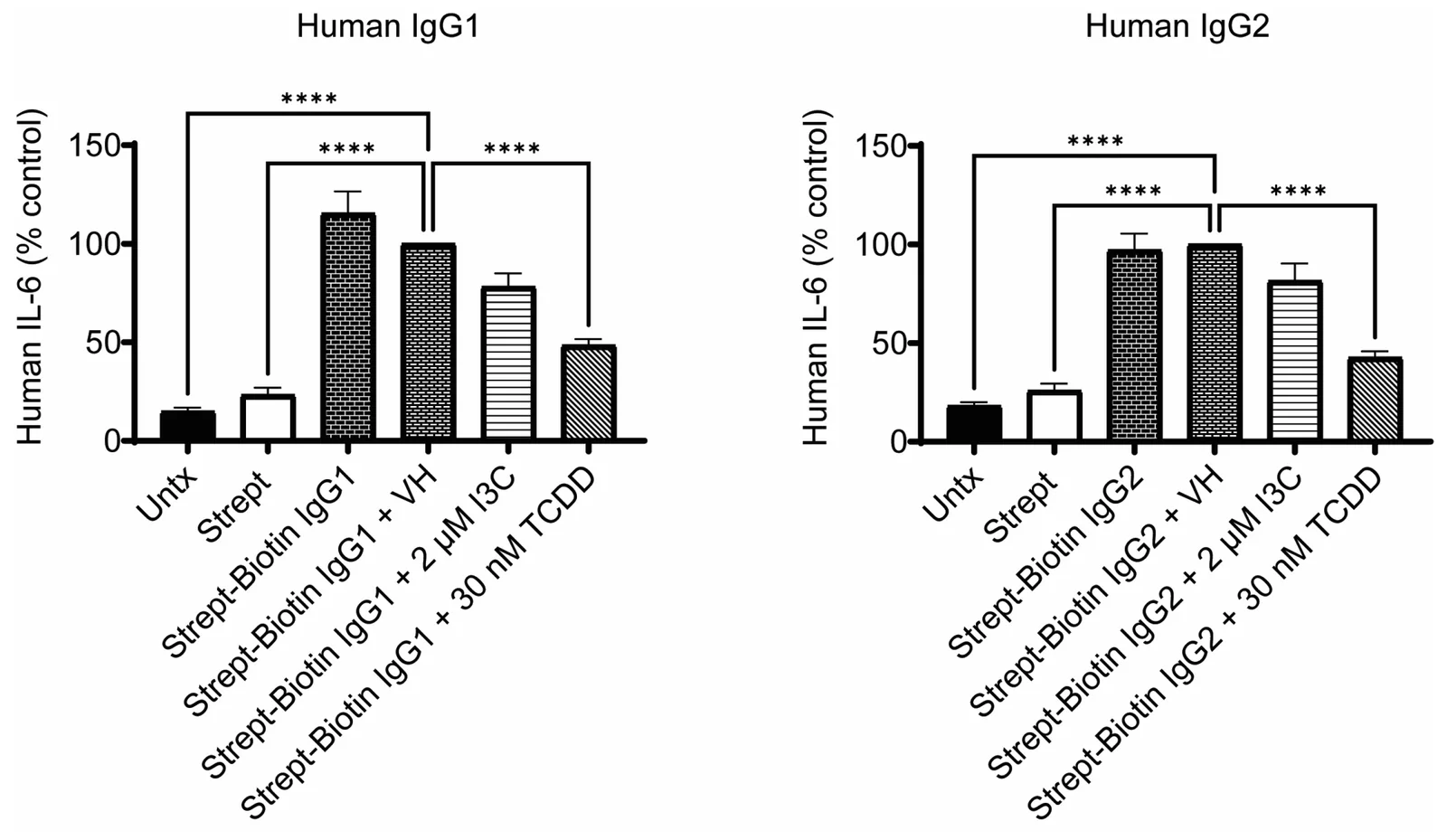
AHR ligand TCDD suppressed Strept-Biotin IgG1 and Strept-Biotin IgG2-mediated IL-6 cytokine secretion. Human PBMCs (mixed sex; sex was not known at time of culture) were plated at 1 × 10^6^ live cells/mL in 100 μL per well. Cells were pretreated with either 0.01% DMSO or AHR ligands: TCDD (30 nM) or I3C (2 µM) in 0.01% DMSO overnight. Cultures were stimulated with either Strept-Biotin IgG1 or Strept-Biotin IgG2 immune complexes the next day and treated again with chemicals for the additional volume from the addition of immune complex and incubated overnight. Supernatants from the culture were assessed for IL-6 cytokine secretion via ELISA. Data obtained in pg/mL were normalized within each donor so data could be combined across donors. Results are from *N* = 24 donors. Before analysis, all datasets were screened for outliers using Grubbs’s test, and a single outlier was removed when detected. Data are mean ± SEM. **** indicates statistical differences compared to the respective IgG complex + VH control at *p* < 0.0001.

To determine if the AHR activation-mediated suppression of antibody production translated into reduced antibody-triggered signaling, human PBMCs were pretreated overnight with VH, TCDD, or I3C on day 0. Cells were stimulated on day 1 with either Strept-Biotin IgG1 or Strept-Biotin IgG2 immune complexes overnight. Stimulation with both Strept-Biotin IgG1 and Strept-Biotin IgG2 immune complexes resulted in a significant increase in IL-6 secretion compared to the Untx and Strept control groups. Interestingly, neither TNF-α nor IL-1β were induced with Strept-Biotin IgG1 and Strept-Biotin IgG2 immune complexes as we have seen with mouse cells [18,19]. Treatment with TCDD significantly suppressed IL-6 production in response to both IgG1 and IgG2 immune complex stimulation. In contrast, I3C produced a modest, but not statistically significant, suppression of IL-6 secretion (Figure 7).

### 3.8. Effects of AHR-Activaing Chemicals on Cell Viability and CD14^+^ Monocyte Cell Population

**Figure 8:**
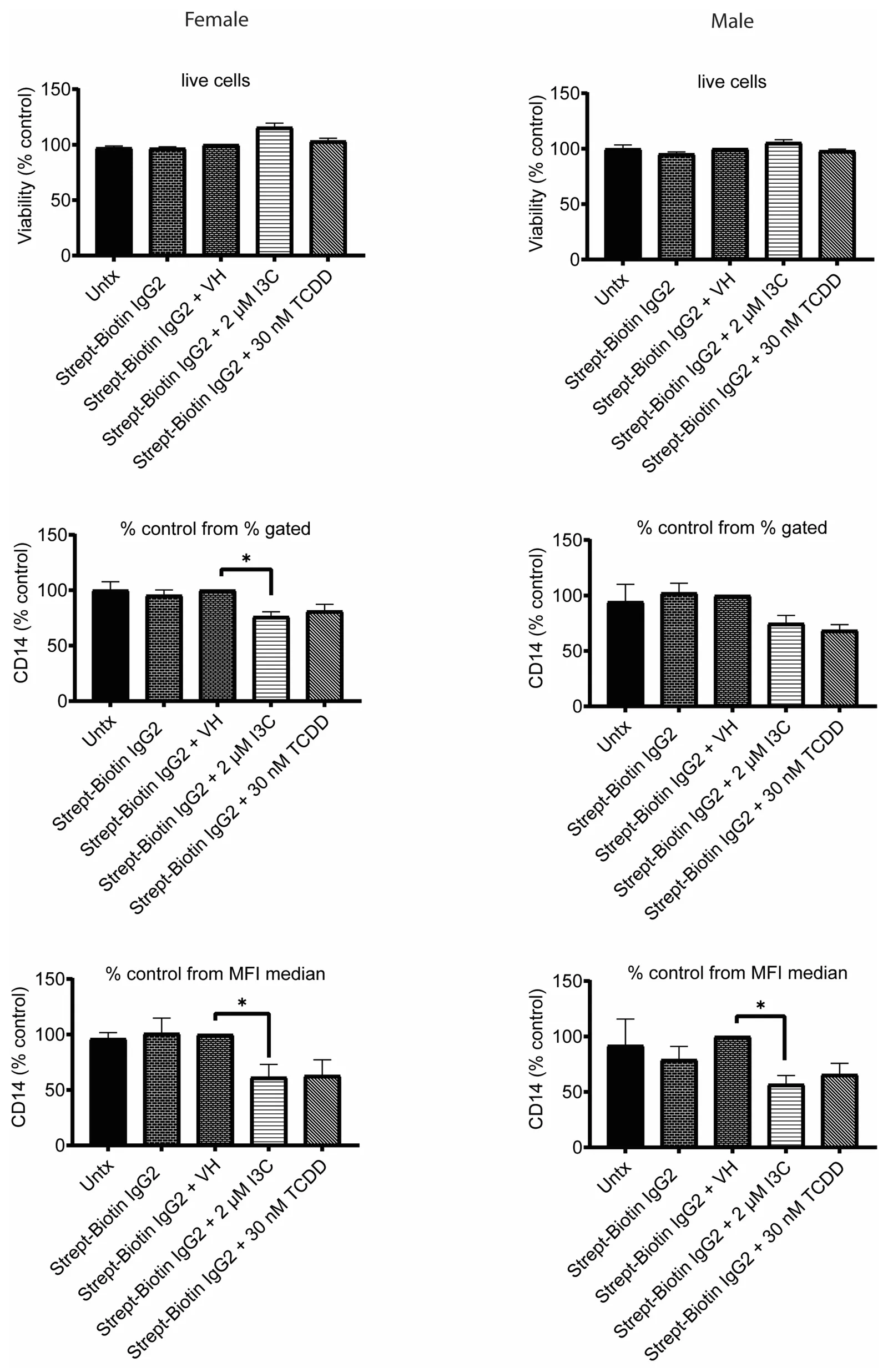
AHR proligand I3C suppressed CD14^+^ expression following Strept-Biotin IgG2 stimulation in female and male human PBMCs without reducing cell viability. Human PBMCs (female or male as noted; sex was known at time of culture) were plated at 1 × 10^6^ live cells/mL in 100 μL per well. Cells were pretreated with either 0.01% DMSO or AHR-activating chemicals: TCDD (30 nM) or I3C (2 µM) in 0.01% DMSO overnight. Cultures were stimulated with Strept-Biotin IgG2 immune complexes the next day and treated again with chemicals for the additional volume from the addition of immune complex and incubated overnight. Cells were stained with Zombie NIR flexible viability kit and CD14. Data obtained (percent gated or MFI median) were normalized within each donor so data could be combined across donors. Results are from *N* = 5 each from female or male donors. Data are mean ± SEM. * indicates statistical differences compared to the Strept-Biotin IgG2 + VH control at *p* < 0.05.

To determine part of the mechanism by which TCDD or I3C might be suppressing IgG-stimulated cytokine production, we assessed viability and CD14^+^ monocyte populations. PBMCs were pretreated with TCDD or I3C, followed by stimulation with Strept-Biotin IgG2 immune complex, and then assessed for cell viability by flow cytometry. Our results showed that neither I3C nor TCDD reduced live-cell viability in cells from female or male donors. Further, we found that I3C significantly reduced the expression of CD14^+^ cells compared to the Strept-Biotin IgG2 + VH control in both male and female PBMCs based on mean fluorescence intensity (MFI; median) and in female PBMCs using percent gated from live singlets. TCDD showed a similar trend but was not statistically significant (Figure 8).

## 4. Discussion

The primary focus of the study was to characterize the mechanisms by which AHR-activating chemicals suppress human IgG antibody production and the potential for AHR activation to produce suppression of IgG-triggered signaling in human PBMCs. Overall, our data demonstrated that AHR-activating chemicals did suppress PWM-stimulated signaling in human PBMCs, although IL-6 production after 2 days of PWM stimulation was more sensitive to suppression by AHR activation than 5 days of PWM-stimulated IgG1. In fact, TCDD was the only AHR ligand that significantly suppressed 2-day PWM-stimulated IL-6, 5-day PWM-stimulated IgG1, and 24 h IgG complex-stimulated IL-6 in human PBMCs. While TCDD reveals some important information regarding the role of AHR in these endpoints, we focused the RNA-seq study on I3C since it represents a non-toxic AHR proligand with potential to exhibit anti-inflammatory and/or immune-suppressive properties [20,21,22]. The GO term and Reactome enrichment analysis revealed that I3C modulated pathways related to cellular signaling, trafficking, and immune regulation. FPKM values revealed that I3C also modulated the AHR transcriptional response, including inducing a significant upregulation of *CYP1A1* and the AHR negative regulator *AHRR*, whereas the expression of *AHR* and *ARNT* remained unchanged with I3C. A targeted analysis of I3C-mediated gene regulation revealed that I3C had only a modest impact on B cell-associated transcription factors, Ig heavy chain genes, inflammatory cytokines, and FcγR expression.

We used PWM to stimulate our cells because it was difficult to stimulate IgG1 production in human PBMCs, which could be due to the fact that IgG1 is already highly expressed in human cells or because we did not pre-purify B cells or naïve B cells initially. We tried several approaches, such as recombinant CD40 ligand (rCD40L) in combination with IL-4, as well as with a combination of rCD40L, IL-4, and IL-21 at various time points; however, our ELISA analysis did not detect any measurable IgG1 production from those stimulations. PWM is a well-established polyclonal mitogen that induces PBMC proliferation and promotes IgG antibody production and has been widely used in in vitro studies to investigate human antibody responses [38,39,40,41,42]. It should be noted that PWM functions primarily as a T cell-dependent activator relying on co-stimulatory interactions between B cells and T cells, inducing T cells to secrete cytokines that are essential for B cell proliferation and differentiation [40,43]. While pure PWM stimulation did not stimulate monocytes directly [44], monocytes might get activated by other cytokines in the PWM-stimulated milieu. Consistent with its role as a potent B cell activator, our results showed that PWM stimulation resulted in a significant upregulation of proinflammatory cytokine gene *IL6* and *IL1B* expression after 2 days of PWM stimulation, although we cannot discern the cell source for the cytokine production from this study. Additionally, B cell differentiation driven by PWM is known to be closely associated with IL-6 signaling [45], because *IL6* mRNA is induced in both T cells and B cells [46]. Thus, we assessed whether activation of AHR signaling could modulate PWM-driven inflammatory immune responses. Our results showed that AHR-activating chemicals, including the high-affinity exogenous ligand TCDD and endogenous or dietary ligands FICZ and ITE, along with the proligand I3C, resulted in a significant reduction in IL-6 secretion following day 2 of PWM stimulation. These findings demonstrate that AHR-activating chemicals, including the non-toxic proligand I3C, can suppress PWM-induced activation of human PBMCs, reducing proinflammatory cytokine production, confirming that AHR activation exerts an immunosuppressive effect on activated human PBMCs.

We focused our RNA-seq analysis on I3C because there is not as much information on I3C as compared to TCDD, and TCDD does not have potential to be developed as a possible therapeutic. Investigating the transcriptomic profile of I3C provides more mechanistic insight into how this non-toxic AHR proligand might modulate immune responses in PWM-stimulated human PBMCs. Although a relatively small number of genes were differentially expressed in the PWM + I3C group as compared to the PWM + VH group, the affected genes and enriched pathways highlight biologically relevant processes associated with immune regulation, intracellular signaling, and cellular organization. Among the downregulated genes, *TRAPPC9* is a regulator of NF-κB activation [23,24], *PLCB1* participates in inflammatory signaling cascades [36,37], and *C1QTNF3* and *WWOX* are involved in immune modulation and inflammatory signaling [28,34,47]. GO term and Reactome enrichment pathways highlighted alterations in GPCR signaling, PLCβ-mediated regulation of inflammation, vesicular trafficking, and organelle biogenesis. While some of the central nervous system/neuronal pathways were unexpected, this might suggest that I3C could be beneficial in neuroinflammation. AHR signaling is known to regulate inflammatory pathways, including NF-κB and cytokine gene expression, to influence immune responses [48,49,50]. *TRAPPC9* plays a role in activation of both the canonical and non-canonical NF-κB pathways [51]. Activation of NF-κB causes the induction of proinflammatory cytokines, including TNF-α, IL-1β, IL-6, chemokines, and additional inflammatory mediators in different types of innate immune cells [52,53]. In addition to *TRAPPC9*, *C1QTNF3* has been shown to activate the MEK/ERK and the PI3K/Akt pathways [54,55,56,57]. PI3K and Akt promote NF-κB activation and subsequent downstream proinflammatory cytokine production [58,59,60]. Given that PI3K/Akt signaling is a known driver of proinflammatory responses, suppression of this signaling pathway would reduce NF-κB activity and attenuate inflammatory cytokine secretion. This suggests that I3C treatment promotes an anti-inflammatory phenotype by downregulation of *TRAPPC9* and *C1QTNF3*. These findings are consistent with our data that showed I3C significantly suppressed PWM-induced proinflammatory IL-6 cytokine secretion. Further, these findings are supported by our previous observations that I3C inhibited Akt phosphorylation induced by both LPS + IFNγ stimulation and mouse Strept-Biotin IgG2b immune complex stimulation in the murine macrophage RAW 264.7 cell line [19].

Next, we analyzed the activation of canonical AHR signaling in human PBMCs by comparing the FPKM value of AHR signaling-related genes. AHR acts as a transcription factor. In the inactive state AHR stays in the cytoplasm with chaperone proteins [61,62,63,64]. Binding to a ligand changes the conformational structure of AHR and leads to dissociation of some chaperone components and allows AHR to translocate into the nucleus where AHR heterodimerizes with its partner protein aryl hydrocarbon receptor nuclear translocator (ARNT). AHR activation results in activation of several metabolizing enzymes such as *CYP1A1* and *CYP1B1* [65,66]. AHR activation also induces its negative regulators *AHRR* and *TIPARP* (also called *PARP7/ARTD14*) to limit AHR-mediated activity [65,67,68]. Our study revealed that expression levels of *AHR* and *ARNT* remained the same with I3C treatment following PWM stimulation. AHR activation was indicated by the significant upregulation of *CYP1A1* and *AHRR*, suggesting that I3C activated the AHR signaling pathway. This indicates that I3C-mediated effects in PWM-stimulated PBMCs were AHR-dependent.

Because we are interested in I3C effects on IgG production and because PWM can stimulate B cells, T cells and likely monocytes, we specifically focused on transcriptomic changes in PWM + I3C on genes associated with B cell function, Ig production, FcγR signaling, and inflammatory responses in human PBMCs, even though these effects were not statistically significant. It should be noted that some of our lack of statistical significance might be due to the fact that the I3C concentration was low as compared to other studies [69,70], or because the degree to which I3C might be converted to its active metabolite, diindolylmethane (DIM), under these conditions in vitro was not quantified. Despite this, our results highlight potential targets and/or sex differences. Our results indicated that I3C modestly downregulated the expression of Ig heavy chain genes, *IGHG1*, *IGHA1*, and *IGHM* consistently in both female and male donors which encode IgG1, IgA1, and IgM, respectively. These finding are consistent with previous research demonstrating that AHR ligands such as TCDD suppress both IgG and IgM antibody secretion in human B cells [8,9,71,72]. This trend is further supported by the downregulation of critical transcription factors involved in B cell differentiation and plasma cell formation, such as *PRDM1*, *XBP1*, *PAX5*, *IRF8*, and *BACH2* in male donors. These observations are consistent with prior reports in mice showing that reduced expression of *Blimp-1*, *Pax-5*, and *Bach2* was associated with compromised IgM response [73,74,75,76], although we did not observe this trend in female donors. Interestingly, I3C modulated FcγR gene expression; *FCGR1A*, *FCGR2A*, and *FCGR2C* receptor expression were upregulated in male donors and *FCGR2C* was upregulated in female donors. As these receptors are primarily activating FcγR [77], this suggests that antibody-triggered response through FcγR could be enhanced with I3C treatment. It should be noted, however, that FcγRs are either activating or inhibitory, and the ultimate effect of I3C on FcγR signaling will be the culmination of effects through all FcγRs. In addition, I3C altered PWM-induced inflammatory cytokine gene expression. PWM stimulation is known to induce a robust proinflammatory response [78], and I3C exhibited immunosuppressive effects in both sexes, although the effected cytokine profile is different in female and male donors. In female donors, *IL2*, *IL10*, *TNF*, *IL1B*, and *IL6* were downregulated, whereas in male donors, *IL4*, *IL10*, *IFNG*, and *IL21* were reduced. Overall, these coordinated changes across B cell regulatory genes, antibody expression, immune signaling and inflammatory response might provide mechanistic insight into how I3C influences PWM-stimulated immune response in human PBMCs, which can be used as a starting point in future studies in which higher concentrations of I3C are investigated. Again, it is important to emphasize that these observations are exploratory, due to the relatively small donor numbers and small changes. Therefore, additional experiments will be necessary at the protein level.

Ultimately, we are interested in investigating a putative link between AHR activation-induced suppression of antibody production from B cells and AHR activation-induced suppression of antibody-triggered signaling in FcγR-expressing cells (including monocytes). As expected, PWM stimulation induced a significant increase in IgG1 production. However, only TCDD reduced the antibody production significantly, again supporting previous immunosuppressive data with TCDD [4,5,6,7,8,9]. In contrast, FICZ, ITE, and I3C did not significantly suppress IgG1 production, although there is evidence that this could be donor-specific. As already noted for I3C, one possible explanation for the lack of a significant effect is that the concentration of the endogenous and dietary ligands used in these experiments might have been insufficient to induce a strong suppressive response though AHR. This would explain our transcriptomic data with PWM + I3C, which revealed only modest changes in gene expression. Another limitation is that we utilized a single time for each endpoint. Although it was selected to precede any IgG protein changes at day 5 post-PWM stimulation, assessing gene changes only at day 2 in response to PWM stimulation might have missed important changes earlier. Interestingly, TCDD also significantly suppressed IL-6 cytokine production following IgG1 and IgG2 immune complex mediated stimulation in human PBMCs, suggesting that TCDD-mediated suppression of IgG antibody production might lead to compromised IgG-triggered signaling. In looking at I3C, again we observe modest non-significant effects, although the trend is toward suppression of IgG-triggered signaling. We noted that the IL-6 suppression was not due to frank cytotoxicity, but that part of the trend of suppression of IL-6 by I3C might involve a decreased CD14^+^ population. This is consistent with other reports of I3C suppressing various cell surface markers in dendritic cells and monocytes [79,80]. Together, these results suggest that antibody-triggered signaling might be a more sensitive target of suppression by AHR-activating chemicals than antibody production itself in human PBMCs.

## 5. Conclusions

Overall, this study demonstrates that I3C exerted immunomodulatory effects in human PBMCs through activation of the AHR pathway. Although I3C did not significantly reduce IgG1 production, it significantly attenuated inflammatory cytokine production that could contribute to compromised antibody production. Transcriptomic analysis revealed I3C mediated modest regulation of B cell function, antibody gene expression, FcγR signaling, and inflammatory responses. However, it also identified potential new targets for I3C, like *TRAPPC9* and *C1QTNF3*, which are involved in immune regulation. Our findings also highlight a correlation between TCDD-mediated suppression of IgG1 and TCDD-mediated suppression of IgG1 (and IgG2)-triggered signaling. Future studies could focus on optimizing the I3C concentration to better define its immunomodulatory potential and the role of its metabolite, DIM. In addition, sex-specific differences in gene regulation and immune responses need further investigation, along with characterizing the functional roles of newly identified targets such as *TRAPPC9* and *C1QTNF3* in mediating AHR-dependent immune modulation. It would also be valuable to identify cell sources of cytokines affected by AHR ligands in response to various stimuli to further delineate the mechanisms of suppression.

## Figures and Tables

**Table 1:** List of downregulated DEGs with I3C treatment following PWM stimulation.

Gene Name	Gene Description	log2FoldChange	Gene Function
*TRAPPC9*	trafficking protein particle complex 9	−1.250431843	Activates NF-κB pathway [23,24]
*SMYD3*	SET and MYND domain containing 3	−1.233663816	Plays an important role in the epigenetic regulation of foxp3 gene, which is essential for iTreg formation [25]
*MSRA*	methionine sulfoxide reductase A	−1.492978211	Reduces methionine sulfoxide residues, protecting cells from oxidative stress [26,27]
*WWOX*	WW domain containing oxidoreductase	−1.705352239	Negative regulator of the noncanonical NF-κB pathway [28]
*SLC9A9*	solute carrier family 9 member A9	−1.573250048	Highly expresses in brain [29,30]
*DOCK3*	dedicator of cytokinesis 3	−1.406876609	Highly expresses in neurons and has a role in neuronal outgrowth [31]
*PRKN*	parkin RBR E3 ubiquitin protein ligase	−5.237543111	Has been suggested to inhibit multiple mechanisms of innate immunity, including mitochondrial antigen presentation [32]
*CPQ*	carboxypeptidase Q	−1.321848515	Serves as an effective gene for M2 macrophage polarization and requitement in case of glioma [33]
*PPM1L*	protein phosphatase, Mg^2+^/Mn^2+^ dependent 1L	−1.109283052	Downregulated apoptosis signal-regulating kinase 1 (ASK1), a protein that initiates a signaling cascade leading to apoptosis when cells are subjected to cytotoxic stress (NCBI; Gene ID: 151742)
*C1QTNF3*	C1q and TNF related 3	−1.385696367	Suppresses TH17 cell differentiation via AdipR2 receptor, inhibits macrophage activation and inflammation [34,35]
*PLCB1*	phospholipase C beta 1	−1.368631052	Crucial regulator in vascular inflammation; silencing it increases the expression of pro-inflammatory cytokines such as IL-1, IL-6, and IL-8 [36]. It is also expressed in macrophages and involved in macrophage-mediated inflammatory response [37]

## Data Availability

The human PBMC PWM + VH and PWM + I3C datasets generated and/or analyzed during the current study are available in the NCBI gene expression omnibus (GEO) GSE329255 (https://www.ncbi.nlm.nih.gov/geo/query/acc.cgi?acc=GSE329255).

## Supplementary Materials

The following supporting information can be downloaded at: https://www.mdpi.com/article/10.3390/antib15040068/s1. Table S1: Raw data used to generate figures and PWM stimulation optimization data.

## Abbreviations

The following abbreviations are used in this manuscript:

AHR	Aryl hydrocarbon receptor
FICZ	6-formylindolo[3,2-b]carbazole
I3C	Indole-3-carbinol
ITE	2-(1H-indol-3-ylcarbonyl)-4-thiazolecarboxylic acid methyl ester
PBMCs	Peripheral blood mononuclear cells
TCDD	2,3,7,8-tetrachlorodibenzo-p-dioxin
